# Basal ganglia and cerebellar interconnectivity within the human thalamus

**DOI:** 10.1007/s00429-016-1223-z

**Published:** 2016-04-18

**Authors:** Esther A. Pelzer, Corina Melzer, Lars Timmermann, D. Yves von Cramon, Marc Tittgemeyer

**Affiliations:** 1 Translational Neurocirciutry Group, Max-Planck Institute for Metabolism Research Cologne, 50931 Cologne, Germany; 2 Department of Neurology, University Clinics Cologne, Cologne, Germany; 3 Max-Planck Institute for Human Cognitive and Brain Sciences, Leipzig, Germany

**Keywords:** Cerebellum, Basal ganglia, Thalamus, Connectivity, Diffusion MRI, Hemispheric lateralization

## Abstract

Basal ganglia and the cerebellum are part of a densely interconnected network. While both subcortical structures process information in basically segregated loops that primarily interact in the neocortex, direct subcortical interaction has been recently confirmed by neuroanatomical studies using viral transneuronal tracers in non-human primate brains. The thalamus is thought to be the main relay station of both projection systems. Yet, our understanding of subcortical basal ganglia and cerebellar interconnectivity within the human thalamus is rather sparse, primarily due to limitation in the acquisition of in vivo tracing. Consequently, we strive to characterize projections of both systems and their potential overlap within the human thalamus by diffusion MRI and tractography. Our analysis revealed a decreasing anterior-to-posterior gradient for pallido-thalamic connections in: (1) the ventral-anterior thalamus, (2) the intralaminar nuclei, and (3) midline regions. Conversely, we found a decreasing posterior-to-anterior gradient for dentato-thalamic projections predominantly in: (1) the ventral-lateral and posterior nucleus; (2) dorsal parts of the intralaminar nuclei and the subparafascicular nucleus, and (3) the medioventral and lateral mediodorsal nucleus. A considerable overlap of connectivity pattern was apparent in intralaminar nuclei and midline regions. Notably, pallidal and cerebellar projections were both hemispherically lateralized to the left thalamus. While strikingly consistent with findings from transneuronal studies in non-human primates as well as with pre-existing anatomical studies on developmentally expressed markers or pathological human brains, our assessment provides distinctive connectional fingerprints that illustrate the anatomical substrate of integrated functional networks between basal ganglia and the cerebellum. Thereby, our findings furnish useful implications for cerebellar contributions to the clinical symptomatology of movement disorders.

**Electronic supplementary material:**

The online version of this article (doi:10.1007/s00429-016-1223-z) contains supplementary material, which is available to authorized users.

## Introduction

Cortex, basal ganglia, cerebellum, and thalamus are interconnected (Alexander and Crutcher 1990; Alexander et al. 1986; Wichmann and DeLong 1996), with basal ganglia and cerebellum being thought to process information in segregated loops that primarily interact in the neocortex. This common understanding has been challenged by findings from neuroanatomical and imaging studies suggesting additionally a direct subcortical interaction with the thalamus as the main relay station of both projection systems (Percheron et al. 1996; Hoshi et al. 2005, suppl. material). Yet, the nature of the reciprocal interactions between cerebellum and basal ganglia is still under debate (Caligiore et al. 2016).

The thalamus receives major projections from the dentate nucleus (DN), which is the main output station of the cerebellum. These projections are ascending via the superior cerebellar peduncle and traverse through as well as anterior to the red nucleus (Asanuma et al. 1983; Ristanović et al. 2010). Conversely, the globus pallidus (GP)—the main output station of the basal ganglia—projects to the thalamus by forming the ansa and fasciculus lenticularis (Gallay et al. 2008). Projections from both systems, from the dentate nucleus as well as from the pallidum, enter the thalamus ventrally by forming the pallido-thalamic and the cerebello-thalamic fascicles, which do not intermingle (Gallay et al. 2008). Both projection systems were so far thought to terminate in segregated territories within the thalamus with DN projections being located posteriorly to the GP projections (Asanuma et al. 1983; Sakai et al. 1996). However, recent studies imply an information exchange already occurring within the thalamus itself (Ichinohe and Shoumura 1998; Bostan et al. 2010, 2013) and by a short latency modulation of the cerebellar output to the basal ganglia (Chen et al. 2014).

To date, information about basal ganglia communication with the cerebellum has been derived by neuroanatomical studies using viral transneuronal tracers in non-human primate brains, developmentally expressed molecular markers (Galvan and Smith 2011; Kuramoto et al. 2011; Penney and Young 1981; Smith et al. 2009, 2014; Ilinsky and Fallet 2004; Nakamura et al. 2012) and studies of pathological human brains (Planetta et al. 2013; Halliday 2009; Rolland et al. 2006; Schmahmann 2003). Hence, our understanding of subcortical basal ganglia and cerebellar interconnectivity within the human thalamus is still sparse due to the obvious limitation of in vivo data acquisition in the intact human brain. So far, only tractography based on diffusion magnetic resonance imaging (dMRI) offers an approach for in vivo analyses of structural connectivity in the human brain (Jbabdi et al. 2015). While a direct inference on construct validity (perhaps by congruence to a gold standard) is still outstanding, current work has augmented evidence on the reproducibility of the dentato-rubro-thalamic tract in post-mortem diffusion tractography (Mollink et al. 2015) and on the robustness of thalamic segmentation based on diffusion tractography (Broser et al. 2011; Traynor et al. 2010).

Until now, a number of human brain studies explored thalamic segmentation with diffusion tractography or based on orientation in dMRI data (e.g., O’Muircheartaigh et al. 2011; Kumar et al. 2015). Within the context of this article, studies specifically focussed on either thalamo-cortical connectivity (Behrens et al. 2003; Johansen-Berg et al. 2005; Klein et al. 2010; O’Muircheartaigh et al. 2015; Seifert et al. 2011), cerebello-thalamic connectivity (Magnotta et al. 2008; Argyelan et al. 2009; Pelzer et al. 2013) or on connectivity patterns in the human basal ganglia per se (including the thalamus, e.g., Draganski et al. 2008; Rozanski et al. 2013; Sharman et al. 2013). The interconnectivity of cortex, basal ganglia, and cerebellum within the thalamus has not yet been comprehensively assessed within the human brain; hence, empirical evidence about territories of connection overlap is lacking.

Analysis of diffusion tractographic data allows for investigating such overlap in connectivity patterns in principle. Although this technique cannot unravel an anatomical distinction between partial interdigitation or local convergence of any two projections in one territory per se, it can facilitate to specify connectional fingerprints per predefined region. This in turn substantiates statistically evaluation of distinct overlaps, thereby elucidating potential functional (or pathophysiological) relevance.

Given potential implications of cerebellar contributions in basal ganglia disorders (Wu and Hallett 2013) and the current lack in mapping thalamic projections within the human brain, we strive to characterize projections of basal ganglia and cerebellum as well as their potential overlap within the human thalamus. We analyzed patterns of pallidal and cerebellar connection territories (1) in relation to histologically predefined thalamic atlases and (2) derive connectional fingerprints as well as probabilistic connectivity maps for an evaluation of basal ganglia and cerebellar interconnectivity.

## Materials and methods

### Data acquisition and subject characteristics

We acquired diffusion-weighted (dMRI) and high-resolution three-dimensional T1-(MPRAGE; TR = 1930 ms, TI = 650 ms, TE = 5.8 ms, resolution 1.0 × 1.0 × 1.25 mm, flip angle 18°, sagittal) and T2- (RARE; TR = 3200 ms, TE = 458 ms, 176 sagittal slices, resolution 1.0 × 1.0 × 1.0 mm^3^) weighted images of 12 right-handed healthy subjects (10 female) on a Siemens 3T Tim TRIO scanner with the subjects understanding and written consent in conformation with the declaration of Helsinki. These subjects were chosen from a larger sample of MRI data that we had acquired recently; we have here chosen only those participants that completely covered the cerebellum in the diffusion scans. Evidently head size plays a role here, and, hence, we sampled predominantly on female participants. All participants included in the study were right-handed (>7th percentile) native German speakers with a mean age of 25 years (range 20–37 years). Participants were screened and excluded if any history or sign of neurological diseases was present. Diffusion-weighted magnetic resonance imaging (dMRI) was performed using echo planar imaging [EPI; TR = 12,000 ms, TE = 100 ms, resolution 1.7 × 1.7 × 1.7 mm^3^, flip angle: 90°, Field of View (FoV): 220 × 220 × 122 mm^3^, bandwidth: 1345 Hz/pixel, orientation: axial, data matrix: 128 × 128 × 72, PAT factor 2, partial Fourier 6/8] with double-spin echo preparation (Reese et al. 2003). Diffusion weighting was isotropically distributed along 60 diffusion-weighted directions (*b* value = 1000 s/mm^2^). Additionally, in each subject seven data sets with no diffusion weighting (b0) were acquired initially and interleaved after each block of 10 diffusion-weighted images as anatomical reference for motion correction. To increase signal-to-noise ratio, scanning was repeated three times for averaging, resulting in a total scan time of approximately 45 min—dMRI data were acquired immediately after the T1- and T2-weighted images in the same scanner reference system.

### Preprocessing

All preprocessing steps were performed with FSL software package (version 4.1.9; http://fsl.fmrib.ox.ac.uk/fsl). First, reorientation of T1-weighted images to the sagittal plane through the anterior and posterior commissures was conducted. The skull was removed from both images applying FSL’s brain extraction tool (BET, for details see Smith 2002). Then the T1-weighted image was used as the individual structural space of each subject and as high-resolution image for further analyses. Subsequently, T1- and T2-images were linearly co-registered and the transformation matrix to diffusion space was calculated using FSL’s registration tool FLIRT (Jenkinson and Smith 2001a) with 12° of freedom. Registration results were controlled visually for every subject. Resulting registration matrices were inverted to allow for mask transformation from diffusion space to structural space for probabilistic tractography. For transformation into standard space, resulting connectivity maps were warped into 1 mm standard Montreal Neurological Institute (MNI) space by the application of FSL’s non-linear registration tool FNIRT (Andersson et al. 2010).

### Outlining of masks

All data sets were controlled for integrity, artifacts, sufficient SNR and homogeneity. Masks of regions of interest (ROI) were outlined on T1- and T2-weighted images with FSL (http://fsl.fmrib.ox.ac.uk/fsl/fslview). ROI-locations were determined based on confirmation with different atlases (Schaltenbrand and Wahren 1977; Mai et al. 2015; Morel 2007; Krauth et al. 2010) after a standardized segmentation protocol, in order to maximize anatomical reliability and minimize inter-individual variability due to uncertainties in ROI-localization. In principle, we defined the whole thalamus (except for the lateral geniculate nucleus) as the seeding region for tractography; DN, representing the main projection source of the deep cerebellar nuclei in humans, and the parts of the pallidum (internal and external part) dorsal to the anterior commissure were chosen as target points (Supplementary Fig. 1, for details cf. Pelzer et al. 2013).

### Probabilistic tractography

FSL’s FDT-toolbox (http://www.fmrib.ox.ac.uk/fsl/fdt/) was applied for probabilistic tractography. To transform seed- and target masks from structural space into the diffusion space, affine transformation matrices that were generated during our preprocessing procedure were implemented into FSL’s probtrackx program (Jenkinson and Smith 2001b); all tractography steps have been performed in diffusion space. Results were afterwards non-linearly transformed to the MNI 152 1 mm standard space for further post-processing and display. The number of samples was *P* = 5000, the number of steps *S* = 2000 with a step length of 0.5 mm; we added a path distribution function in order to correct for differences in distance between seed and target regions. The curvature threshold was: *c* = 0.2 (corresponding to a minimum angle of approximately ±80°, see http://fsl.fmrib.ox.ac.uk/fsl/fslwiki/FDT/-UserGuide#PROBTRACKX_probabilistic_tracking_with_crossing_fibres).

### Connectivity measure

Considering all fibres originating in a given seed region S, its structural connectivity with a given target region T can be defined in terms of the proportion of those fibres that intersect T while running within the brain white matter, yielding a number in the interval between 0 (no fibres intercept T) and 1 (all fibres starting in S reach T). This quantity (*φ*) gives no information about the absolute number of connections between two regions, but reflects the degree of connectivity or relative connectivity density. It can be considered as a measure of the likelihood of a connection in the sense that it can be interpreted as the frequency at which one would reach T by randomly seeding a fiber starting within S. In our framework, the notions of anatomical connection strength and anatomical connection likelihood are therefore inter-changeable (for a more detailed discussion of this issue, cf. Stephan et al. 2009).

**Fig. 1:**
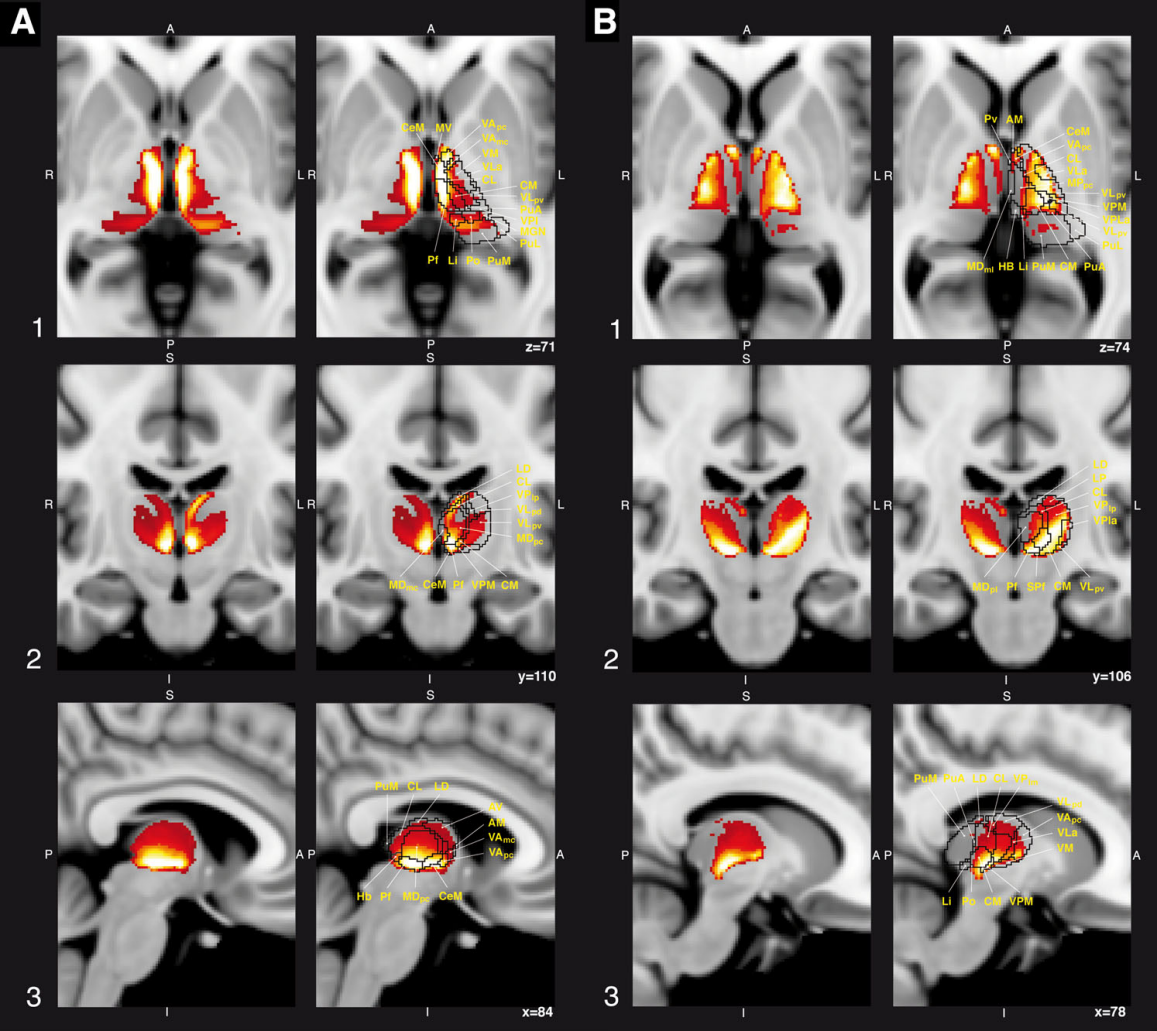
Connection strength (*φ*) of pallido-thalamic (**a**) and dentate-thalamic projections (**b**). High and low connectivity is represented in a *color scale* ranging from *yellow* to *red*, respectively, where *yellow* denotes highly connected and *red* low connectivity. Within the thalamus the pallidal projection territory is located primarily in anterior and medial regions, whereas the dentate projection territory is located more laterally and posterior. The medial thalamic region and the intralaminar nuclei are overlapping zones for both territories

From this, connectivity values were derived by a normalization of the geometric mean of the robust maxima of each individual connectivity map. The normalized connectivity maps were warped to MNI 1 mm standard space; subsequently the arithmetic mean of all normalized maps was determined for further analysis. To minimize noise, we threshold connection probabilities to include only those voxels higher than the lower 95 % confidence interval (CI). Visual evaluation was enabled by masking the resulting probability maps with histologically identified thalamic nuclei provided by Krauth et al. (2010). Taken from this atlas, a 3D volume of all thalamic sub-nuclei (as enlisted in Table 1) was overlaid onto the connectivity map separately for each projection (pallidal or cerebellar, respectively) and the results were slice-by-slice evaluated in 2D along the *x*, *y* and *z* axis. The relative connectivity density was caught in a continuous color scale, where high and low connectivity is yellow and red, respectively (Fig. 1). Evidently the three-dimensional reconstruction of a cytoarchitectonically based thalamic atlas can be just an approximation of an in vivo anatomical thalamic territory. We have chosen this atlas primarily, because it provides a standard and was provided in the 1 mm MNI-152 space. We displayed probabilities for connectivity in FSLVIEW (http://fsl.fmrib.ox.ac.uk/fslview). No further hard segmentation procedure to classify these connectivity maps was performed in order to sustain streamline distributions even at low connection probabilities. Atlas-based thalamic “sub-territories” followed the revised Anglo-American nomenclature (see Table 1, as well as Hirai and Jones 1989).

**Table 1:** Connotation of thalamic “sub-territories” as implemented in the three-dimensional atlas of the human thalamus, after Krauth et al. (2010)

Name	Abbreviation	Name	Abbreviation
Anterodorsal Nucleus	AD	Posterior nucleus	Po
Anteromedial nucleus	AM	Anterior pulvinar	PuA
Anteroventral nucleus	AV	Inferior pulvinar	Pul
Central medial nucleus	CeM	Lateral pulvinar	PuL
Central lateral nucleus	CL	Parvalbumin	PV
Center médian nucleus	CM	Suprageniculate nucleus	SG
Fasciculus cerebello-thalamicus	fct	Nucl. Tegmenti pedunculopontinus, pars compacta	TPP
Habenular nucleus	Hb	Ventral anterior nucleus	VA
Lateral dorsal nucleus	LD	Ventral anterior nucleus (magnocellular divisions)	VAmc
Lateral geniculate nucleus (magnocellular layers)	LGNmc	Ventral anterior nucleus (parvocellular divisions)	VApc
Lateral geniculate nucleus (parvocellular layers)	LGNpc	Ventral lateral nucleus	VL
Limitans nucleus	Li	Ventral lateral anterior nucleus	VLa
Lateral posterior nucleus	LP	Ventral lateral posterior nucleus	VLp
Mediodorsal nucleus	MD	Ventral lateral posterior nucleus (dorsal division)	VLpd
Mediodorsal nucleus (parvocellular division)	MDpc	Ventral lateral posterior nucleus (ventral division)	VLpv
Mediodorsal nucleus (magnocellular division)	MDmc	Ventral medial nucleus	VM
Medial geniculate nucleus	MGN	Ventral posterior inferior nucleus	VPI
Mamillothalamic tract	Mtt	Ventral posterior lateral nucleus	VPL
Medioventral nucleus	MV	Ventral posterior lateral nucleus (anterior divisions)	VPLa
Parafascicular nucleus	Pf	Ventral posterior lateral nucleus (posterior divisions)	VPLp
Subparafascicular nucleus	sPf	Ventral posterior medial nucleus	VPM
Medial pulvinar	PuM

### Statistical analysis

**Fig. 2:**
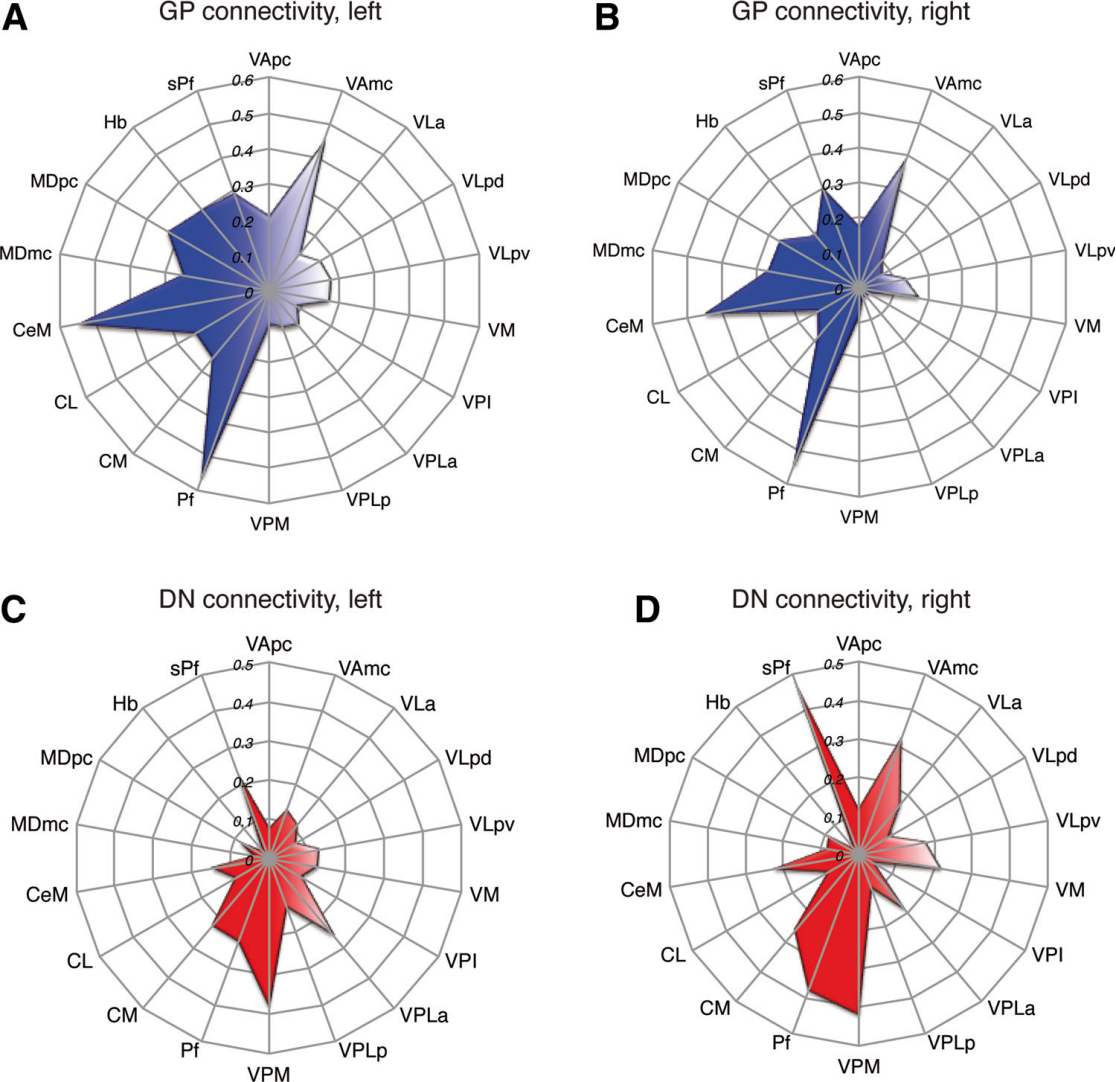
Connectivity fingerprints for pallido-thalamic and cerebello-thalamic projections. The *values* indicate the relative connection density for pallidal (in the **a**, *left*, and, **b**, right hemisphere) or cerebellar (**c**, *left*; **b**, *right*) connectivity with thalamic sub-territories (for the connotation of different nuclei, please cf. Krauth et al. 2010, or Table 1)

**Fig. 3:**
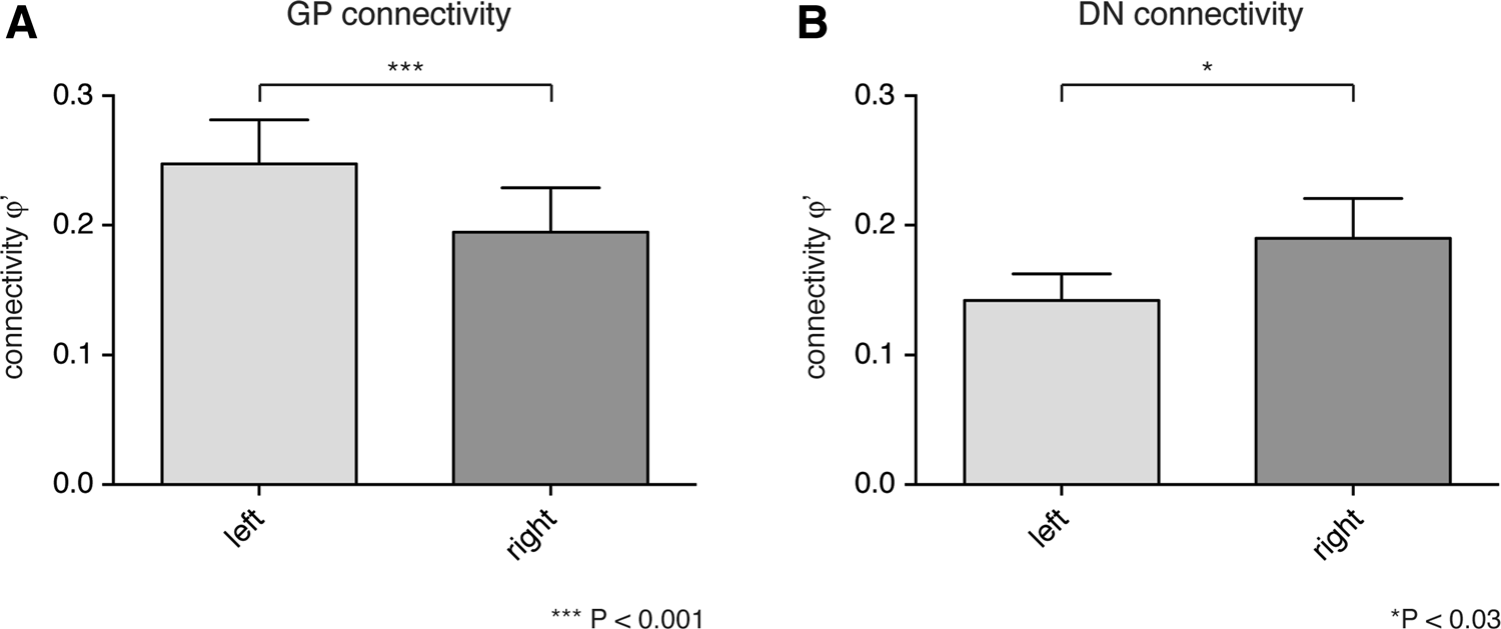
Analysis of the hemispheric difference in pallidal and cerebellar connection density within the thalamus. Overall connectivity values (mean ± SEM) were higher from GP to thalamus in the *left* hemisphere than in the *right* (**a**, *p* < 0.001), whereas projections from right DN into the left hemispheric thalamus were lower compared to connectivity values for projections from left DN into the thalamus of the right hemisphere (**b**, *p* < 0.05). All regions that have been considered for the connectional fingerprints (Fig. 2) were included in the analysis

After anatomical evaluation, relative connection strength was estimated for each thalamic territory by applying the individual nuclei territory maps provided by Krauth et al. (2010). To select connectivity values associated with each thalamic territory, we took a robust average per subject. Statistical outliers were excluded by the robust regression and outlier removal (ROUT) method with a threshold of false discoveries of *Q* = 2 % [where *Q* determines how aggressively the method will remove outliers; we took a fairly conservative approach (Motulsky and Brown 2006)]. We then normalized individual connectivity values for pallidal and cerebellar connection probability to the maximum connectivity value of the left and right hemisphere individually; herewith the size in the seed or target region was taken into account to render the results comparable. Connectivity fingerprints were then derived for each projection pattern (Fig. 2). Relative connectivity densities (*φ*) of the sub-territories were tested in a Wilcoxon-matched pair-sign-rank test between the left and right hemisphere due to the nonparametric distribution revealed by a D’Agostino and Pearson omnibus normality test (*p* < 0.05; Fig. 3).

## Results

### Pallido-thalamic connectivity

The map of pallido-thalamic projections had a stratified appearance with peak connectivity in medial regions and regions of VA (Fig. 1a). Within a 95 % confidence interval, connectivity densities were higher in the left hemisphere as compared to the right hemisphere. A gradient decreasing along the antero-posterior extent was found in VA/VL regions: inferior and anterior parts of VA revealed the highest connection strength. Reasonably high connectivity revealed for antero-medial parts of VLp, for VM, as well as for dorsal and anterior parts of VPM; to a lesser extent anterior and medial parts of VLa were connected with the pallidum. More medially, the caudal group nuclei of the intralaminar nuclei, were highly connected, specifically the whole Pf and anterior parts of sPf, and to a considerably lesser degree the anteromedial parts of the CM. All dorsal group nuclei (CL and CeM) were strongly connected. In the medial nuclei group inferior parts of MDpc, lateral parts of MDmc and the medioventral nucleus were highly connected; and, additionally, anterior parts of the epithalamus, i.e., the Habenla (Hb). In the anterior thalamic area, posterior parts of LD were highly connected; in the lateral posterior group, dorsal and posterior parts, i.e., LP, expressed high connectivity. Antero- and dorso-medial parts of the pulvinar and PuM had medium connection strength.

All remaining regions exhibited no or only very moderate probability for pallidal projections. The mammillothalamic tract could be clearly identified and passed the pallidal territory with low connection probability.

In an assessment of the connection densities, we focussed on ventral, medial and intralaminar thalamic nuclei as representative regions, because these have been previously described to be main territories of pallidal projections (for results of remaining territories, cf. Suppl. Figure 2 A/B). The connectivity of different sub-territories for pallidal projections was characterized by territory-specific ‘connectional fingerprint’ for the left (Fig. 2a) and right hemisphere (Fig. 2b), respectively. Within this fingerprints, the general pattern was highly congruent between both hemispheres, yet the overall connectivity for all pallidal projections in the considered sub-territories was significantly different between both hemispheres (*p* < 0.001; see Fig. 3a).

### Dentato-thalamic connectivity

In contrast to findings following pallido-thalamic tractography, connectivity with the DN indicate a gradient decreasing along the postero-anterior extent, which is particularly pronounced in ventral thalamic nuclei (Fig. 1b).

Highest connection strength was deduced for ventroposterior nuclei regions of the thalamus, comprising dorsal and anterior parts of VPLa, ventral and anterior parts of VPLp, as well as VPM. The other ventral nuclei (including VM, VLa and VLpv) revealed less high but still pronounced connectivity values; the dorsal and posterior parts of VLpd were connected with considerably lower probability. Inferior and posterior parts of VApc and anterior and posterior parts of VAmc were again highly connected. Anterior regions of VPI were only moderately connected on the left side, but not on the right.

The intralaminar nuclei were highly connected in ventral parts of CM, dorsal and ventral parts of CeM, ventral parts of Pf, and sPf, as well as in antero-medial and dorsal parts of CL.

The medial division of the dorsal thalamus, MV and lateral parts of MDpc, are highly connected; especially in the right hemisphere MDmc density of connectivity values is low. Moreover, the anterior division of the dorsal thalamus, comprising posterior parts of AV and AM, were highly connected with DN; left and anterior parts of the LD exhibited high connectivity in the right hemisphere, but none was found in the left hemisphere.

Anterior and inferior parts of PuA were only moderately connected. No or only low connectivity values of dentate-thalamic projections were found for the remaining thalamus.

In an assessment of the connection densities, we again focussed on ventral, medial and intralaminar thalamic nuclei as representative regions that have also been characterized to be main territories of cerebellar projections (Sakai et al. 1996; for results of remaining territories, cf. Suppl. Figure 2 C/D). Territory-specific connectional fingerprints for dentatal projections were estimated separately for the right (Fig. 2c) and left hemisphere (Fig. 2d). Here, the general pattern was again highly congruent between both hemispheres, yet it appeared not as similar as for the pallidal projections in an inter-hemispheric comparison. Still the overall connectivity differed significantly between the right and left hemisphere (*p* < 0.05); here, higher connection strength’ from the right dentate nucleus to the left thalamus were found (see Fig. 3b).

## Discussion

We assessed pallido-thalamic and dentato-thalamic connectivity for specific thalamic sub-territories. As transneuronal tracing is not assessable in humans, information on these connectivity aspects until now resides on the studies from the expression of molecular markers or pathological brain studies. Therefore, we used tractography based on diffusion MRI for our evaluation. While this type of technique does not comprehensibly allow to differentiate every anatomical detail in projection overlaps per se (see below), it facilitates two prominent aspects of connectivity information (Jbabdi et al. 2015): (1) the qualitative appraisal of fiber distributions and (2) the quantitative assessment of relative connection densities. Suchlike connection density of both subcortical systems and their potential overlap can be characterized and statistically substantiated within the human thalamus.

Comparisons of the interconnectivity of projections from the cerebellum and basal ganglia have been a matter of intensive discussion in the anatomical literature (see, e.g., Jones 2007). Especially reports on projections to the human thalamus bear numerous discrepancies and open questions, most prominently with regard to the conflict of mismatch between afferent and efferent projections and due to vast differences in thalamic nomenclature (Krack et al. 2002); when considering data from diffusion tractography as well, there might arise an additional issue with respect to the different spatial resolution and anatomical aperture.

Pioneers as Vogt and Vogt (1920) already described pallidal connections with the (motor) thalamus, e.g., the VA/VL complex, in humans. They used diverse methods to describe connections of the striatal system, the pallidum and the thalamus (here morphological descriptions and interpretations from myeloarchitecture, cytoarchitecture and histology, Vogt and Vogt (1941)). Their results were replicated in following decades by studies in non-human primates (Carpenter and Strominger 1967; Kim et al. 1976; Parent and De Bellefeuille 1982; Fénelon et al. 1990; Sakai et al. 1996; Kuo and Carpenter 2004; Nauta and Mehler 1966), in rats (Carter and Fibiger 1978), and in cats (Larsen and Sutin 1978; Harnois and Filion 1982). But also studies with molecular markers like GABA and glutamate (Kuramoto et al. 2011; Penney and Young 1981; Bosch-Bouju et al. 2013) and studies of pathological brains (Schmahmann 2003; Rolland et al. 2006; Halliday 2009) supported a territory specific distribution.

In agreement with our findings, regions such as VM were determined as part of the pallido-thalamic projection territory (Kultas-Ilinsky et al. 1980; Haroian et al. 1981)—this was conclusively reported also in human post-mortem studies (Gallay et al. 2008). Also in congruency with our results, only few pallidal projections reach VPL (Sakai et al. 1996). A discrepancy, however, occurs by our finding that seems to suggest only moderate pallidal connectivity with VLa. This may be due to the specific intra-pallidal fiber organization and associated with a problem of diffusion tractography to resolving this: From anatomical tracing studies, dorsal parts of the internal segment of the pallidum (GPi) prominently project to VApc by passing VM; conversely, ventral GPi projections are rather expected in posterior regions of the thalamus, such as VLa (Kuo and Carpenter 2004). In our study, we restricted ourselves to include only GP segments dorsal to the anterior commissure in our mask (see Methods), the ventral pallidum was therefore disregarded (cf. Suppl. Figure 1). This decision may have biased connection densities in VLa.

Connections to the dorsal (CL; CeM) and caudal intralaminar nuclei (CM; Pf) have been described to be the main target for efferent (Nauta and Mehler 1966; Sakai et al. 1996; Sidibe et al. 1997; Kuo and Carpenter 2004; Sadikot and Rymar 2009) as well as afferent (Kincaid et al. 1991; Yasukawa et al. 2004; Sadikot and Rymar 2009) pallidal projections, in line with our findings. Moreover, from our studies projections in medial thalamic regions—including MDpc, lateral parts of MDmc and anterior parts of the Hb—seem highly probable, which is also in line with existing animal studies (MD, e.g., Mitchell and Chakraborty (2013); Hb, e.g. Parent and De Bellefeuille (1982)).

Additionally, we found pallidal projection zones in dorsal thalamic areas—in posterior parts of LD, dorsal and posterior parts of LP and anterior and dorsal parts of PuM. Projections to the lateral aspects have been described in rats (Sakai and Bruce 2004), and only very moderate projections to the medial pulvinar (PuM) are in accordance to existing studies as well: Although afferent projection from GP to the pulvinar have been reported (DeVito et al. 1980), PuM is the thalamic nucleus with a distinguished overlap of thalamocortical but only few subcortical projections (Murray and Wallace 2011).

Cerebellar projections to VA/VL regions have been intensively studied (Stanton 1980; Kalil 1981; Asanuma et al. 1983; Percheron et al. 1996; Middleton and Strick 2000), suggesting dense projections from the deep cerebellar nuclei (mainly DN) to VLp.; these projections include postero-dorsal parts and antero-medial parts of VLp, as well as regions between the islands of VLa (Asanuma et al. 1983; Percheron et al. 1996; Middleton and Strick 2000). In human post-mortem studies, entrance of cerebellar fibres was demonstrated in the ventral division of VLpv, with a possible extension into VLa (Gallay et al. 2008). Caudal region of VA was described to be part of the cerebellar projection field in accordance with our findings (Kusama et al. 1971; Stanton 1980; Kalil 1981)—although controversial discussions about these projections exist (Asanuma et al. 1983; Ilinsky and Kultas-Ilinsky 1987; Percheron et al. 1996). Cerebellar afferents to the VM, as reported in our study (yet without the possibility to impute direction of information transfer), were repeatedly confirmed after lesions in the brachium conjunctivum (Kultas-Ilinsky et al. 1980; Haroian et al. 1981; Gallay et al. 2008). In the ventro-posterior thalamic area, cerebellar projections ascending within the cerebello-thalamic fascicule that pass VPM on their way to VLp are well described (Gallay et al. 2008). Next to dorsal column-lemniscal afferents into the VPLa, cerebellar axons pass the somesthetic nuclei of VPM (Asanuma et al. 1983; Percheron et al. 1996). Ascending projections of DN entering VPI have been confirmed in a study in the monkey (Sakai et al. 1996), but just as ‘fibres en passage’ without termination—these findings are also in line to our findings. The posterior division of VPL was, as in our study, no main zone of cerebellar projections; however, in our study small inferior and anterior parts of VPLp were highly connected. This may be due to incongruences between histologically defined atlas-based regions and, hence, a matter of resolution.

Regarding the intralaminar nuclei, each of the deep cerebellar nuclei projects to dorsal—i.e., to CL (Kalil 1981; Asanuma et al. 1983; Sakai et al. 1996; Sadikot and Rymar 2009) or CeM (Haroian et al. 1981)—and caudal intralaminar nuclei—i.e., to CM and inferior parts of Pf (Sakai and Patton 1993; Sakai et al. 1996; Gallay et al. 2008), which we could confirm in our analysis (Fig. 1b). Excitatory projections to the subparafascicular nucleus, as part of the mesodiencephalic junction, are well described and match to our findings (De Zeeuw et al. 1998); notably also fibres ‘en passage’, like suprarubral fibres from the dentato-thalamic tract, may have additively enhanced the connectivity values in this small nucleus; it is, however, methodologically not possible to differentiate between terminal fields and fibres ‘en passage’ by diffusion tractography. Also well in line with our results are findings that assign the territory of MD as a projection territory of the cerebellum (Middleton and Strick 2000). To our knowledge, cerebellar projections to anterior thalamic nuclei or LD have not yet been directly investigated by transneuronal tracing studies, but an involvement of the cerebellum with the Papez circuit had been formerly considered (Snider and Maiti 1976). Cerebellar projections passing the pulvinar have been documented in the monkey and might support moderate connection probabilities in our analysis (Stanton 1980).

**Fig. 4:**
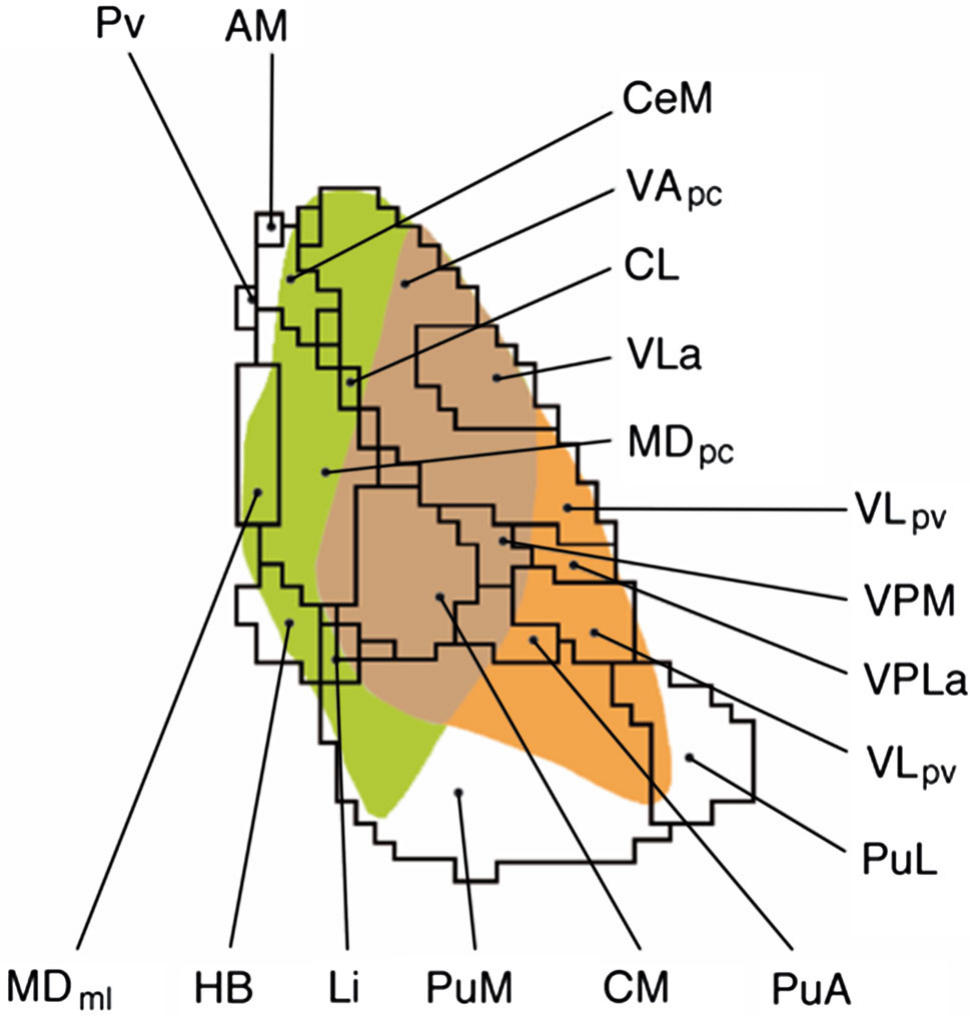
Schematic overview of pallido-thalamic and dentato-thalamic projections. Pallido-thalamic projections (*green*) are located more anteriorly and medially than dentate-thalamic projections (*orange*). Although both projection systems indicate a particular territory specific accentuation, overlapping regions exists (such as the VLa or the CM/PF-complex, for instance)

The main organization principle in the thalamus with respect to both, pallidal and cerebellar, projections, is a segregation of terminal fields (Middleton and Strick 2000). In the current anatomical literature, different regions of potential overlap, such as VA, VL or VM, the intralaminar nuclei or the medial nuclei group have been examined (e.g., Sakai et al. 1996; Rouiller et al. 1994). To which extent, however, these projection systems really segregate, converge or interdigitate has been discussed controversially (e.g., Percheron et al. 1996; Ilinsky and Kultas-Ilinsky 1987). It is a matter of dispute whether, rather than serving as a means for widespread cortical areas to gain access to the motor system, the recipient thalamic neurons of the multiple, possibly segregated, loops perform other functions, like cognition (Middleton and Strick 2000). The targets within the medial (intralaminar) areas and within the lateral area might be related to segregated streams (as postulated from the cerebellum) and might convey cerebellar input to either the striatum or the cortex, respectively, and subserving different functions (Caligiore et al. 2016). Our analysis will not resolve this debate completely. The applied technique, diffusion tractography, cannot in principle differentiate converging synapses from interdigitating projection patterns, and it does neither lead to discriminate direction of information transfer nor to determine an axonal termination, but it greatly holds promise to define thalamic territories that are likely for projection overlaps. In this context, we visualized our findings for the regions discussed above (Fig. 4), what might stimulate the above debate.

Furthermore, our analysis revealed a lateralization of pallido-thalamic connectivity in the left thalamus (*p* < 0.001; see Fig. 3a) and a lateralization of the projections from the right cerebellar hemisphere to the left thalamus (*p* < 0.05; see Fig. 3b).

This asymmetry in connectivity likely reflects the functional hemispheric specialization, such as proposed by Stephan et al. (2007). That is, evidently two distinct forms of functional lateralization are present in the left vs the right cerebral hemisphere (Gotts et al. 2013). These may correlate with accentuated projections accordingly of individual thalamic nuclei.

In this context, a well-known finding is a dominance pattern in basal ganglia motor activation (Scholz et al. 2000): the left basal ganglia nuclei seem more active than the right for right handers, regardless of hand use. Alike a functional lateralization has been reported for the also cerebellum (Bernard et al. 2014; Mattay et al. 1998; Schlerf et al. 2015), in accordance to our findings. Yet our analyses also point towards lateralization in connectivity beyond the motor domain. We find predominantly pallidal and cerebellar connections to be lateralized in medial thalamic regions (esp. the medial and intralaminar nuclei) as well as in VAmc. Hence, underlying function might rather be constrained to non-motor domains (Van der Werf et al. 2002). As medial and intralaminar nuclei each receive specific sets of afferents and project to parts of the cerebral cortex and basal ganglia, a lateralization in these thalamic subgroups does likely also exist. Moreover, the targets of the thalamo-cortical and thalamo-basal ganglia projections of a given nucleus are interconnected through cortico-basal ganglia projections. Therefore, the medial and intralaminar nuclei might have a dual role in cortico-subcortical interactions in the forebrain; interestingly, output station from the thalamus to the forebrain is the VAmc region, which is densely connected to medial and intralaminar regions (Carmel 1970).

In basal ganglia disorders, such as Parkinson’s disease, structural and functional alterations of the thalamus have been described (Rolland et al. 2006; Planetta et al. 2013; Halliday 2009). Recent pathophysiological concepts consider a compensatory function of the cerebellum during initial disease stages, and a further modulatory role of cerebellar nuclei on basal ganglia circuitry during disease progression (Wu and Hallett 2013). Here, the thalamus is regarded as the main relay station of both projection systems. Given these implications and the current lack in mapping thalamic projections within the human brain, clearly further analysis is needed to elucidate pathophysiological hypotheses on basal ganglia disease progression as well as to enrich concepts for operative treatment of movement disorders. In this context, diffusion tractography will complement further neuroanatomical studies by contributing in vivo findings.

In conclusion, pallidal and cerebellar projections to the thalamus show a territory specific organization with (1) segregated areas and (2) overlapping regions. Interestingly, these overlapping regions are remarkably larger than it has been described earlier in animal studies. Although the underlying anatomical distinction of the overlap cannot be dissolved by diffusion tractography, our findings highlight the possibility for a larger information exchange between basal ganglia and cerebellum at thalamic level. These findings may thereby also indicate the increasing compensatory role of the cerebellum in basal ganglia disorders like Parkinson’s disease.

## Electronic supplementary material

Below is the link to the electronic supplementary material.

Supplementary material 1 (PDF 1107 kb)

## References

[CR1] Alexander GE, Crutcher MD (1990). Functional architecture of basal ganglia circuits: neural substrates of parallel processing. Trends Neurosci.

[CR2] Alexander GE, DeLong MR, Strick PL (1986). Parallel organization of functionally segregated circuits linking basal ganglia and cortex. Annu Rev Neurosci.

[CR3] Andersson JLR, Jenkinson M, Smith S (2010) Non-linear registration, aka spatial normalization. FMRIB Analysis Group of the University of Oxford, FMRIB technical report TR07JA2

[CR4] Argyelan M, Carbon M, Niethammer M, Ulug AM, Voss HU, Bressman SB, Dhawan V, Eidelberg D (2009). Cerebellothalamocortical connectivity regulates penetrance in dystonia. J Neurosci.

[CR5] Asanuma C, Thach W, Jones E (1983). Distribution of cerebellar terminations and their relation to other afferent terminations in the ventral lateral thalamic region of the monkey. Brain Res Rev.

[CR6] Behrens TEJ, Johansen Berg H, Woolrich MW, Smith SM, Wheeler-Kingshott CAM, Boulby PA, Barker GJ, Sillery EL, Sheehan K, Ciccarelli O, Thompson AJ, Brady JM, Matthews PM (2003). Non-invasive mapping of connections between human thalamus and cortex using diffusion imaging. Nat Neurosci.

[CR7] Bernard JA, Peltier SJ, Benson BL, Wiggins JL, Jaeggi SM, Buschkuehl M, Jonides J, Monk CS, Seidler RD (2014). Dissociable functional networks of the human dentate nucleus. Cereb Cortex.

[CR8] Bosch-Bouju C, Hyland BI, Parr-Brownlie LC (2013). Motor thalamus integration of cortical, cerebellar and basal ganglia information: implications for normal and parkinsonian conditions. Front Comput Neurosci.

[CR9] Bostan AC, Dum RP, Strick PL (2010). The basal ganglia communicate with the cerebellum. Proc Natl Acad Sci U S A.

[CR10] Bostan AC, Dum RP, Strick PL (2013). Cerebellar networks with the cerebral cortex and basal ganglia. Trends Cogn Sci.

[CR11] Broser P, Vargha-Khadem F, Clark CA (2011). Robust subdivision of the thalamus in children based on probability distribution functions calculated from probabilistic tractography. Neuroimage.

[CR12] Caligiore D, Pezzulo G, Baldassarre G, Bostan AC, Strick PL, Doya K, Helmich RC, Dirkx M, Houk J, Jörntell H, Lago-Rodriguez A, Galea JM, Miall RC, Popa T, Kishore A, Verschure PFMJ, Zucca R, Herreros I (2016) Consensus Paper: Towards a Systems-Level View of Cerebellar Function: the Interplay Between Cerebellum, Basal Ganglia, and Cortex. Cerebellum, Ahead of Print. doi: papers3://publication/doi/10.1007/s12311-016-0763-3 10.1007/s12311-016-0763-3 PMC5243918 26873754

[CR13] Carmel PW (1970). Efferent projections of the ventral anterior nucleus of the thalamus in the monkey. Am J Anat.

[CR14] Carpenter MB, Strominger NL (1967). Efferent fibers of the subthalamic nucleus in the monkey. A comparison of the efferent projections of the subthalamic nucleus, substantia nigra and globus pallidus. Am J Anat.

[CR94] Carter D, Fibiger H (1978). The projections of the entopeduncular nucleus and globus pallidus in rat as demonstrated by autoradiography and horseradish peroxidase histochemistry. J Comp Neurol.

[CR15] Chen CH, Fremont R, Arteaga-Bracho EE, Khodakhah K (2014). Short latency cerebellar modulation of the basal ganglia. Nat Neurosci.

[CR16] De Zeeuw CI, Simpson JI, Hoogenraad CC, Galjart N, Koekkoek SK, Ruigrok TJ (1998). Microcircuitry and function of the inferior olive. Trends Neurosci.

[CR17] DeVito JL, Anderson ME, Walsh KE (1980). A horseradish peroxidase study of afferent connections of the globus pallidus in Macaca mulatta. Exp Brain Res.

[CR18] Draganski B, Kherif F, Kloeppel S, Cook PA, Alexander DC, Parker GJM, Deichmann R, Ashburner J, Frackowiak RSJ (2008). Evidence for segregated and integrative connectivity patterns in the human basal ganglia. J Neurosci.

[CR19] Fénelon G, François C, Percheron G, Yelnik J (1990). Topographic distribution of pallidal neurons projecting to the thalamus in macaques. Brain Res.

[CR20] Gallay MN, Jeanmonod D, Liu J, Morel A (2008). Human pallidothalamic and cerebellothalamic tracts: anatomical basis for functional stereotactic neurosurgery. Brain Struct Func.

[CR21] Galvan A, Smith Y (2011). The primate thalamostriatal systems: anatomical organization, functional roles and possible involvement in Parkinson’s disease. Basal Ganglia.

[CR22] Gotts SJ, Jo HJ, Wallace GL, Saad ZS, Cox RW, Martin A (2013). Two distinct forms of functional lateralization in the human brain. Proc Natl Acad Sci U S A.

[CR23] Halliday GM (2009). Thalamic changes in Parkinson’s disease. Parkinsonism Relat Disord.

[CR96] Harnois C, Filion M (1982). Pallidofugal projections to thalamus and midbrain: a quantitative antidromic activation study in monkeys and cats. Exp Brain Res.

[CR24] Haroian AJ, Massopust LC, Young PA (1981). Cerebellothalamic projections in the rat: an autoradiographic and degeneration study. J Comp Neurol.

[CR25] Hirai T, Jones EG (1989). A new parcellation of the human thalamus on the basis of histochemical staining. Brain Res Rev.

[CR26] Hoshi E, Tremblay L, Féger J, Carras PL, Strick PL (2005). The cerebellum communicates with the basal ganglia. Nat Neurosci.

[CR27] Ichinohe N, Shoumura K (1998). A di-synaptic projection from the superior colliculus to the head of the caudate nucleus via the centromedian-parafascicular complex in the cat: an anterograde and retrograde labeling study. Neurosci Res.

[CR28] Ilinsky KK, Fallet C (2004). Development of the human motor-related thalamic nuclei during the first half of gestation, with special emphasis on GABAergic circuits. J Comp Neurol.

[CR29] Ilinsky IA, Kultas-Ilinsky K (1987). Sagittal cytoarchitectonic maps of the Macaca mulatta thalamus with a revised nomenclature of the motor-related nuclei validated by observations on their connectivity. J Comp Neurol.

[CR30] Jbabdi S, Sotiropoulos SN, Haber SN, Van Essen DC, Behrens TE (2015). Measuring macroscopic brain connections in vivo. Nat Neurosci.

[CR31] Jenkinson M, Smith S (2001). A global optimisation method for robust affine registration of brain images. Med Image Anal.

[CR32] Jenkinson M, Smith S (2001). A global optimisation method for robust affine registration of brain images. Med Image Anal.

[CR33] Johansen-Berg H, Behrens TEJ, Sillery E, Ciccarelli O, Thompson AJ, Smith SM, Matthews PM (2005). Functional-anatomical validation and individual variation of diffusion tractography-based segmentation of the human thalamus. Cereb Cortex.

[CR34] Jones EG (2007). The thalamus.

[CR35] Kalil K (1981). Projections of the cerebellar and dorsal column nuclei upon the thalamus of the rhesus monkey. J Comp Neurol.

[CR36] Kim R, Nakano K, Jayaraman A, Carpenter MB (1976). Projections of the globus pallidus and adjacent structures: an autoradiographic study in the monkey. J Comp Neurol.

[CR37] Kincaid AE, Penney JB, Young AB, Newman SW (1991). The globus pallidus receives a projection from the parafascicular nucleus in the rat. Brain Res.

[CR38] Klein JC, Rushworth MF, Behrens TE, Mackay CE, de Crespigny AJ, D’Arceuil H, Johansen-Berg H (2010). Topography of connections between human prefrontal cortex and mediodorsal thalamus studied with diffusion tractography. Neuroimage.

[CR39] Krack P, Dostrovsky J, Ilinsky I, Kultas-Ilinsky K, Lenz F, Lozano A, Vitek J (2002). Surgery of the motor thalamus: problems with the present nomenclatures. Mov Disord.

[CR40] Krauth A, Blanc R, Poveda A, Jeanmonod D, Morel A, Székely G (2010). A mean three-dimensional atlas of the human thalamus: generation from multiple histological data. Neuroimage.

[CR41] Kultas-Ilinsky K, Ilinsky IA, Young PA, Smith KR (1980). Ultrastructure of degenerating cerebellothalamic terminals in the ventral medial nucleus of the cat. Exp Brain Res.

[CR42] Kumar V, Mang S, Grodd W (2015). Direct diffusion-based parcellation of the human thalamus. Brain Struct Func.

[CR43] Kuo JS, Carpenter MB (2004). Organization of pallidothalamic projections in the rhesus monkey. J Comp Neurol.

[CR44] Kuramoto E, Fujiyama F, Nakamura KC, Tanaka Y, Hioki H, Kaneko T (2011). Complementary distribution of glutamatergic cerebellar and GABAergic basal ganglia afferents to the rat motor thalamic nuclei. Eur J Neurosci.

[CR45] Kusama T, Mabuchi M, Sumino T (1971). Cerebellar projections to the thalamic nuclei in monkeys. Proc Japan Acad.

[CR95] Larsen KD, Sutin J (1978). Output organization of the feline entopeduncular and subthalamic nuclei. Brain Res.

[CR46] Magnotta VA, Adix ML, Caprahan A, Lim K, Gollub R, Andreasen NC (2008). Investigating connectivity between the cerebellum and thalamus in schizophrenia using diffusion tensor tractography: a pilot study. Psychiatry Res.

[CR47] Mai JK, Majtanik M, Paxinos G (2015). Atlas of the human brain.

[CR48] Mattay VS, Callicott JH, Bertolino A, Santha AK, Van Horn JD, Tallent KA, Frank JA, Weinberger DR (1998). Hemispheric control of motor function: a whole brain echo planar fMRI study. Psychiatry Res.

[CR49] Middleton FA, Strick PL (2000). Basal ganglia and cerebellar loops: motor and cognitive circuits. Brain Res Rev.

[CR50] Mitchell AS, Chakraborty S (2013). What does the mediodorsal thalamus do?. Front Syst Neurosci.

[CR51] Mollink J, van Baarsen KM, Dederen PJWC, Foxley S, Miller KL, Jbabdi S, Slump CH, Grotenhuis JA, Kleinnijenhuis M, van Cappellen van Walsum AM (2015) Dentatorubrothalamic tract localization with postmortem MR diffusion tractography compared to histological 3D reconstruction. Brain Struct Func, Ahead of Print. doi:10.1007/s00429-015-1115-7 10.1007/s00429-015-1115-7 PMC5009171 26438333

[CR52] Morel A (2007). Stereotactic atlas of the human thalamus and basal ganglia.

[CR53] Motulsky HJ, Brown RE (2006). Detecting outliers when fitting data with nonlinear regression—a new method based on robust nonlinear regression and the false discovery rate. BMC Bioinform.

[CR54] Murray MM, Wallace MT (2011) The neural bases of multisensory processes. CRC Press, Boca Raton, FL 22593873

[CR55] Nakamura KC, Sharott A, Magill PJ (2012). Temporal coupling with cortex distinguishes spontaneous neuronal activities in identified basal ganglia-recipient and cerebellar-recipient zones of the motor thalamus. Cereb Cortex.

[CR56] Nauta WJ, Mehler WR (1966). Projections of the lentiform nucleus in the monkey. Brain Res.

[CR57] O’Muircheartaigh J, Vollmar C, Traynor C, Barker GJ, Kumari V, Symms MR, Thompson P, Duncan JS, Koepp MJ, Richardson MP (2011). Clustering probabilistic tractograms using independent component analysis applied to the thalamus. Neuroimage.

[CR58] O’Muircheartaigh J, Keller SS, Barker GJ, Richardson MP (2015). White matter connectivity of the thalamus delineates the functional architecture of competing thalamocortical systems. Cereb Cortex.

[CR59] Parent A, De Bellefeuille L (1982). Organization of efferent projections from the internal segment of globus pallidus in primate as revealed by flourescence retrograde labeling method. Brain Res.

[CR60] Pelzer EA, Hintzen A, Goldau M, von Cramon DY, Timmermann L, Tittgemeyer M (2013). Cerebellar networks with basal ganglia: feasibility for tracking cerebello-pallidal and subthalamo-cerebellar projections in the human brain. Eur J Neurosci.

[CR61] Penney JB, Young AB (1981). GABA as the pallidothalamic neurotransmitter: implications for basal ganglia function. Brain Res.

[CR62] Percheron G, François C, Talbi B, Yelnik J, Fénelon G (1996). The primate motor thalamus. Brain Res Rev.

[CR63] Planetta PJ, Schulze ET, Geary EK, Corcos DM, Goldman JG, Little DM, Vaillancourt DE (2013). Thalamic projection fiber integrity in de novo Parkinson disease. Am J Neuroradiol.

[CR64] Reese TG, Heid O, Weisskoff RM, Wedeen VJ (2003). Reduction of eddy-current-induced distortion in diffusion MRI using a twice-refocused spin echo. Magn Reson Med.

[CR65] Ristanović D, Milošević NT, Stefanović BD, Marić DL, Rajković K (2010). Morphology and classification of large neurons in the adult human dentate nucleus: a qualitative and quantitative analysis of 2D images. Neurosci Res.

[CR66] Rolland A-S, Herrero M-T, Garcia-Martinez V, Ruberg M, Hirsch EC, François C (2006). Metabolic activity of cerebellar and basal ganglia-thalamic neurons is reduced in parkinsonism. Brain.

[CR67] Rouiller EME, Liang FF, Babalian AA, Moret VV, Wiesendanger MM (1994). Cerebellothalamocortical and pallidothalamocortical projections to the primary and supplementary motor cortical areas: a multiple tracing study in macaque monkeys. J Comp Neurol.

[CR68] Rozanski VE, Vollmar C, Cunha JP, Tafula SMN, Ahmadi S-A, Patzig M, Mehrkens J-H, Bötzel K (2013). Connectivity patterns of pallidal DBS electrodes in focal dystonia: a diffusion tensor tractography study. Neuroimage.

[CR69] Sadikot AF, Rymar VV (2009). The primate centromedian-parafascicular complex: anatomical organization with a note on neuromodulation. Brain Res Bull.

[CR70] Sakai ST, Bruce K (2004). Pallidothalamocortical pathway to the medial agranular cortex in the rat: a double labeling light and electron microscopic study. Thalamus Relat Syst.

[CR71] Sakai ST, Patton K (1993). Distribution of cerebellothalamic and nigrothalamic projections in the dog: a double anterograde tracing study. J Comp Neurol.

[CR72] Sakai S, Inase M, Tanji J (1996). Comparison of cerebellothalamic and pallidothalamic projections in the monkey (Macaca fuscata): a double anterograde labeling study. J Comp Neurol.

[CR73] Schaltenbrand G, Wahren W (1977). Atlas for stereotaxy of the human brain.

[CR74] Schlerf JE, Galea JM, Spampinato D, Celnik PA (2015). Laterality differences in cerebellar-motor cortex connectivity. Cereb Cortex.

[CR75] Schmahmann JD (2003). Vascular syndromes of the thalamus. Stroke.

[CR76] Scholz VH, Flaherty AW, Kraft E, Keltner JR, Kwong KK, Chen YI, Rosen BR, Jenkins BG (2000). Laterality, somatotopy and reproducibility of the basal ganglia and motor cortex during motor tasks. Brain Res.

[CR77] Seifert S, von Cramon DY, Imperati D, Tittgemeyer M, Ullsperger M (2011). Thalamocingulate interactions in performance monitoring. J Neurosci.

[CR78] Sharman M, Valabregue R, Perlbarg V, Marrakchi-Kacem L, Vidailhet M, Benali H, Brice A, Lehéricy S (2013). Parkinson’s disease patients show reduced cortical-subcortical sensorimotor connectivity. Mov Disord.

[CR79] Sidibe M, Bevan MD, Bolam JP, Smith Y (1997). Efferent connections of the internal globus pallidus in the squirrel monkey: I. Topography and synaptic organization of the pallidothalamic projection. J Comp Neurol.

[CR80] Smith SM (2002). Fast robust automated brain extraction. Hum Brain Mapp.

[CR81] Smith Y, Raju D, Nanda B, Pare J-F, Galvan A, Wichmann T (2009). The thalamostriatal systems: anatomical and functional organization in normal and parkinsonian states. Brain Res Bull.

[CR82] Smith Y, Galvan A, Ellender TJ, Doig N, Villalba RM, Huerta-Ocampo I, Wichmann T, Bolam JP (2014). The thalamostriatal system in normal and diseased states. Front Syst Neurosci.

[CR83] Snider RS, Maiti A (1976). Cerebellar contributions to the Papez circuit. J Neurosci Res.

[CR84] Stanton GB (1980). Topographical organization of ascending cerebellar projections from the dentate and interposed nuclei in Macaca mulatta: an anterograde degeneration study. J Comp Neurol.

[CR85] Stephan KE, Fink GR, Marshall JC (2007). Mechanisms of hemispheric specialization: insights from analyses of connectivity. Neuropsychologia.

[CR86] Stephan KE, Tittgemeyer M, Knösche TR, Moran RJ, Friston KJ (2009). Tractography-based priors for dynamic causal models. Neuroimage.

[CR87] Traynor C, Heckemann RA, Hammers A, O’Muircheartaigh J, Crum WR, Barker GJ, Richardson MP (2010). Reproducibility of thalamic segmentation based on probabilistic tractography. Neuroimage.

[CR88] Van der Werf YD, Witter MP, Groenewegen HJ (2002). The intralaminar and midline nuclei of the thalamus. Anatomical and functional evidence for participation in processes of arousal and awareness. Brain Res Rev.

[CR89] Vogt C, Vogt O (1920). Zur Lehre der Erkrankungen des striatären systems. J Psychol Neurol.

[CR90] Vogt C, Vogt O (1941). Thalamusstudien I-III. J Psychol Neurol.

[CR91] Wichmann T, DeLong MR (1996). Functional and pathophysiological models of the basal ganglia. Curr Opin Neurobiol.

[CR92] Wu T, Hallett M (2013). The cerebellum in Parkinson’s disease. Brain.

[CR93] Yasukawa TT, Kita TT, Xue YY, Kita HH (2004). Rat intralaminar thalamic nuclei projections to the globus pallidus: a biotinylated dextran amine anterograde tracing study. J Comp Neurol.

