# Supplementary material for: Basal ganglia and cerebellar interconnectivity within the human thalamus
Source: Brain Struct Funct. 2016 Apr 18;222(1):381–92. doi: 10.1007/s00429-016-1223-z (PMC5225161; doi:10.1007/s00429-016-1223-z)
Supplement: Supplementary file 1 — Supplementary material 1 (PDF 1107 kb) [file 429_2016_1223_MOESM1_ESM.pdf]

SUPPLEMENTARY MATERIAL

S1 Seed and target mask outline used for tractography

Figure S1: Example of seed and target masks, which were included in the tractography analysis. GP: Globus pallidus (internal and external part); Thal: Thalamus; DN: Dentate nucleus

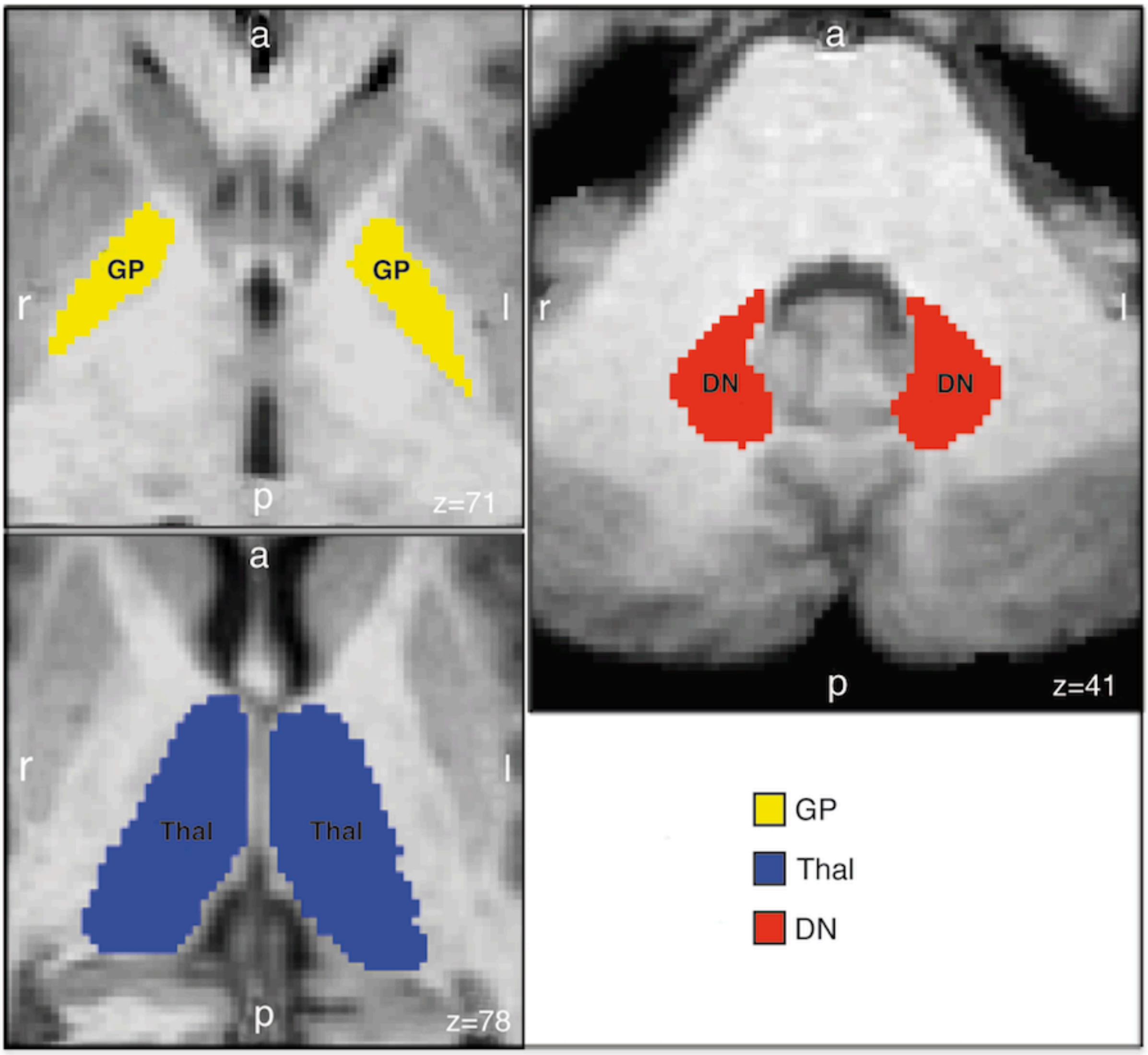

S2 Connectivity fingerprints for pallido-thalamic and cerebello-thalamic projections

Figure 2: Connectional fingerprints for all thalamic projections not revealed in Figure 2. The values indicate the relative connection density for pallidal (in the A, left, and B, right hemisphere) or cerebellar (C, left; B, right) connectivity with thalamic sub-territories (for the connotation of different nuclei, please cf. Krauth et al., 2010, or Table 1).

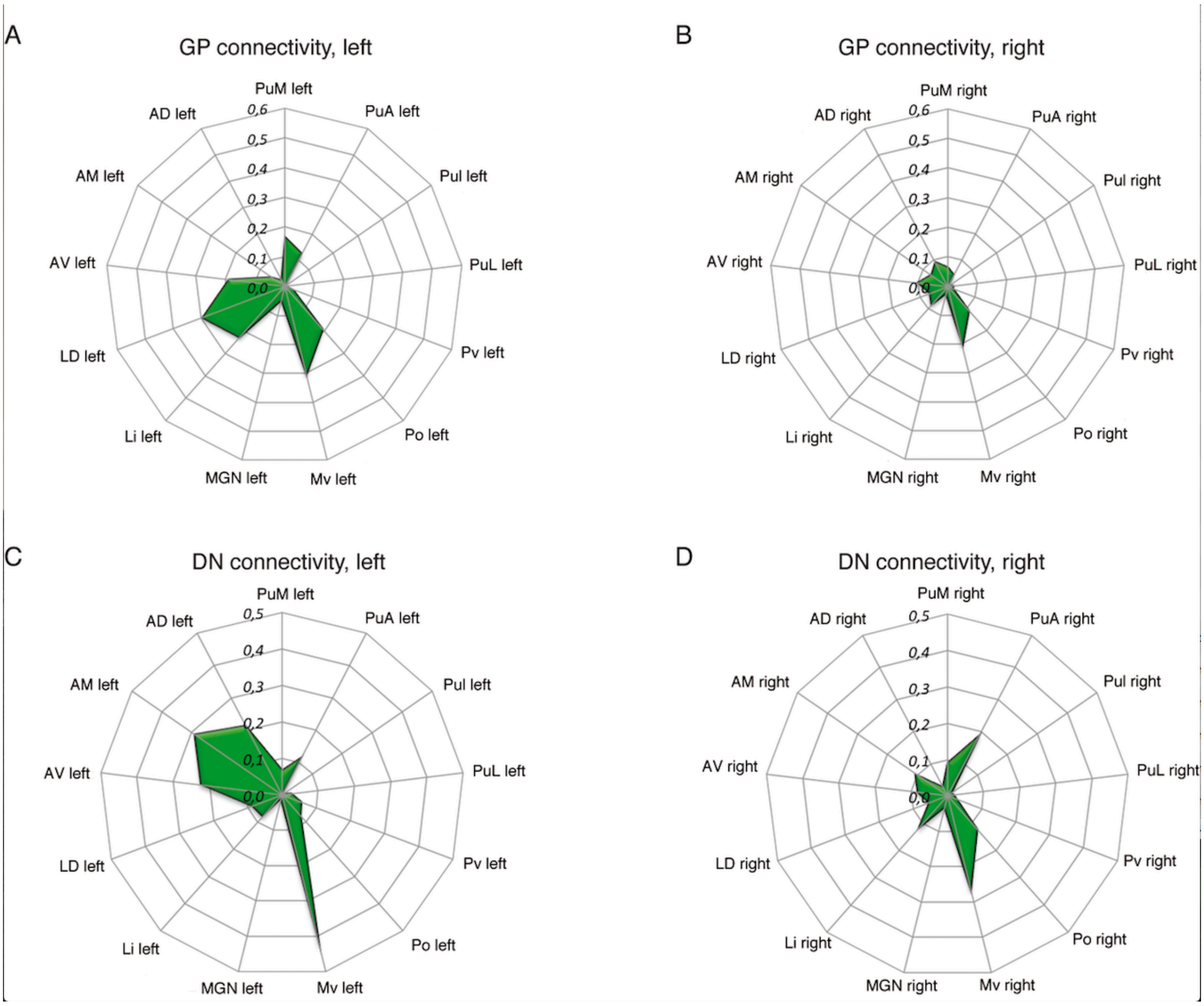
A
GP connectivity, left
PuM left
PuA left
Pul left
PuL left
Pv left
Po left
Mv left
MGN left
Li left
LD left
AV left
AM left
AD left
0,0
0,1
0,2
0,3
0,4
0,5
0,6
B
GP connectivity, right
PuM right
PuA right
Pul right
PuL right
Pv right
Po right
Mv right
MGN right
Li right
LD right
AV right
AM right
AD right
C
DN connectivity, left
D
DN connectivity, right
